# Supplementary figures and images for: RET enhancer haplotype-dependent remodeling of the human fetal gut development program
Source: PLoS Genet. 2023 Nov 10;19(11):e1011030. doi: 10.1371/journal.pgen.1011030 (PMC10664930; doi:10.1371/journal.pgen.1011030)

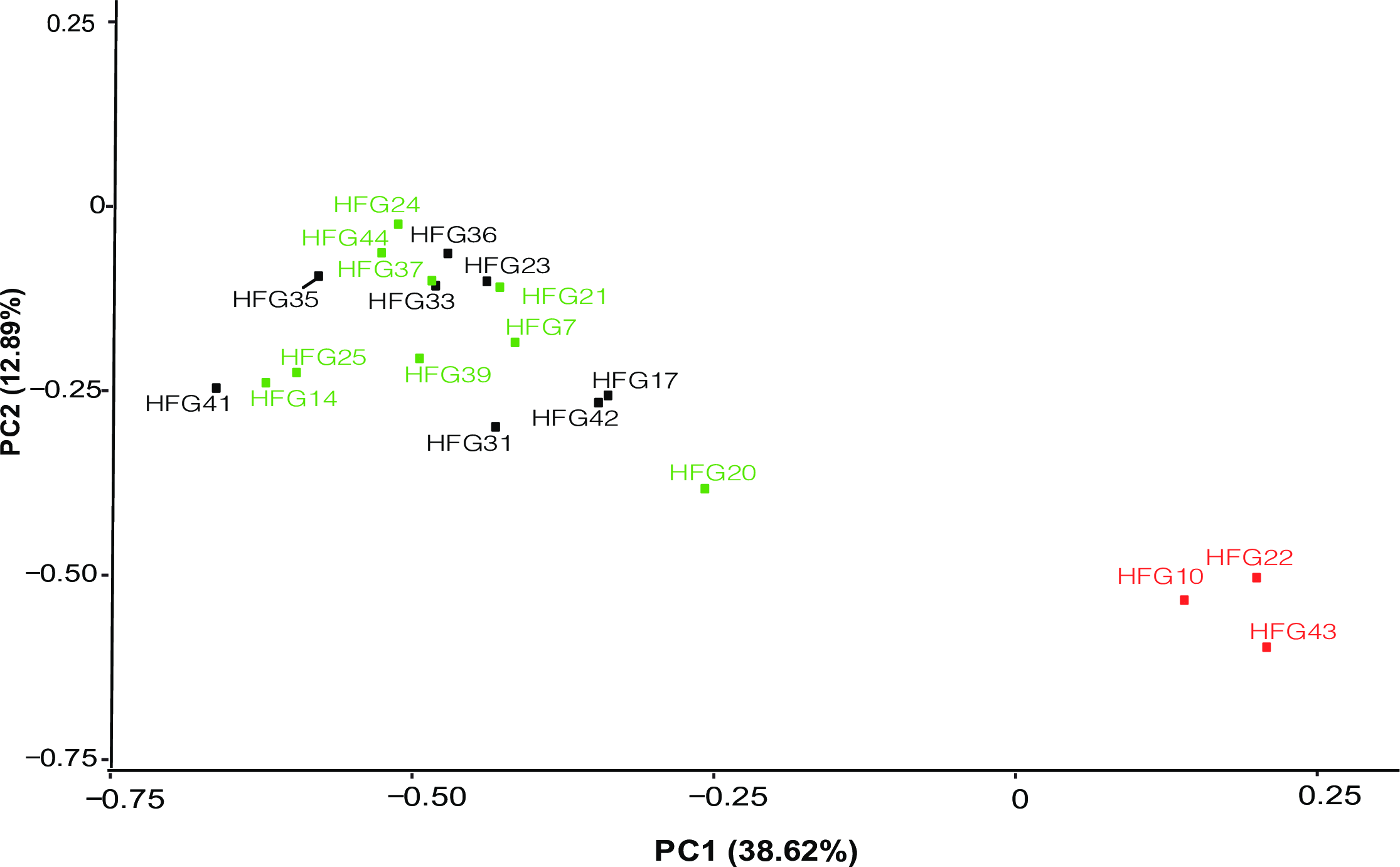

Supplement: S1 Fig — The principal component analysis on all expressed genes demonstrates clustering of RR (green) and RS samples (black) together, reflective of their similar gene expression patterns which is very different from the SS samples (red). (TIF) [file pgen.1011030.s004.tif]

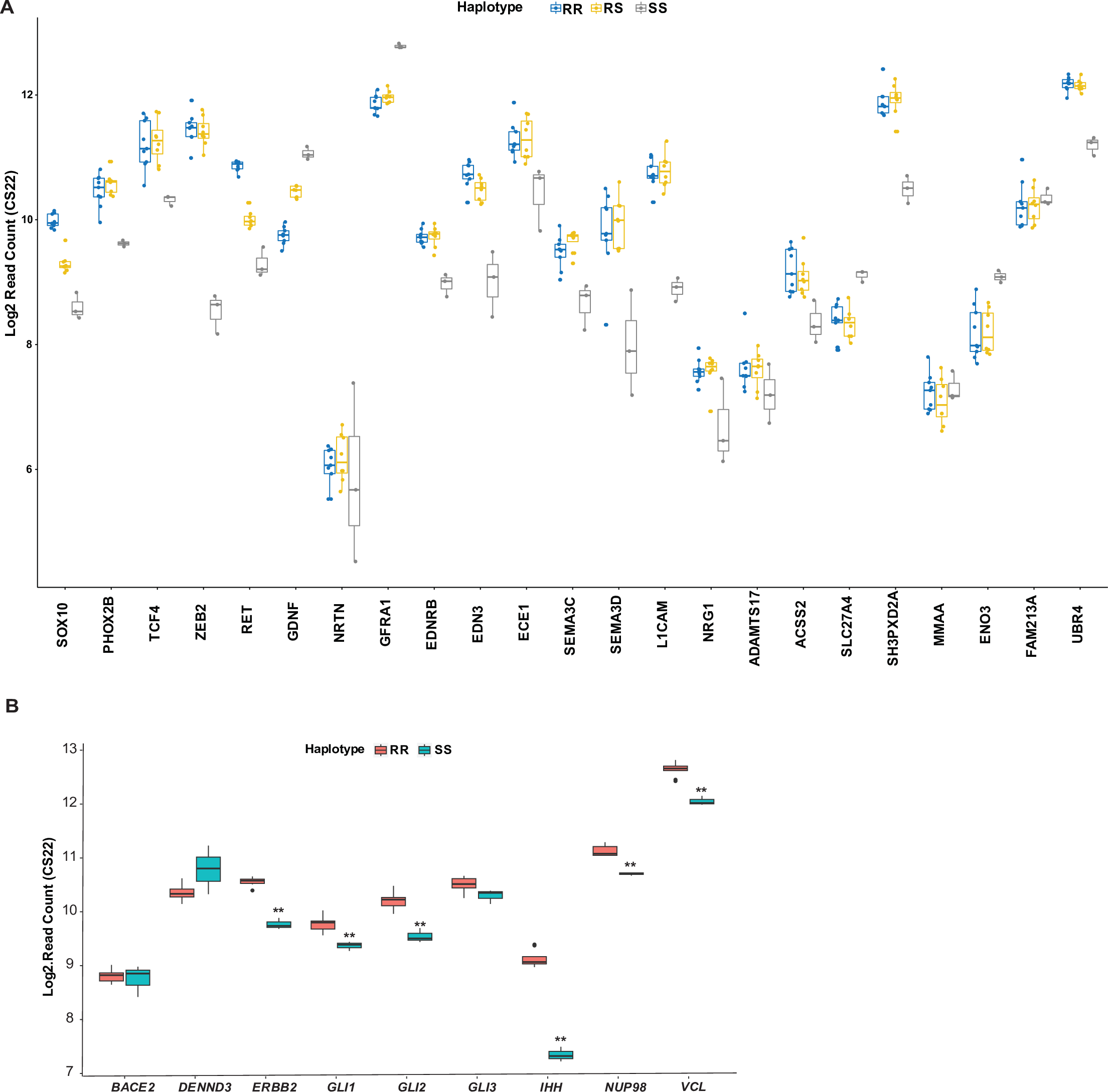

Supplement: S2 Fig — (A) Gene expression differences between RR, RS and SS genotypes at 24 HSCR genes, demonstrates very similar expression of these genes between RR and RS samples. (B) Analysis of gene expression changes of between RR and SS samples for 9 newly discovered HSCR associated genes detects significantly lower expression of ERBB2, GLI1, GLI2, IHH, NUP98 and VCL in SS haplotype (*, **: Benjamini-Hochberg FDR < 0.01, 0.001). (TIF) [file pgen.1011030.s005.tif]

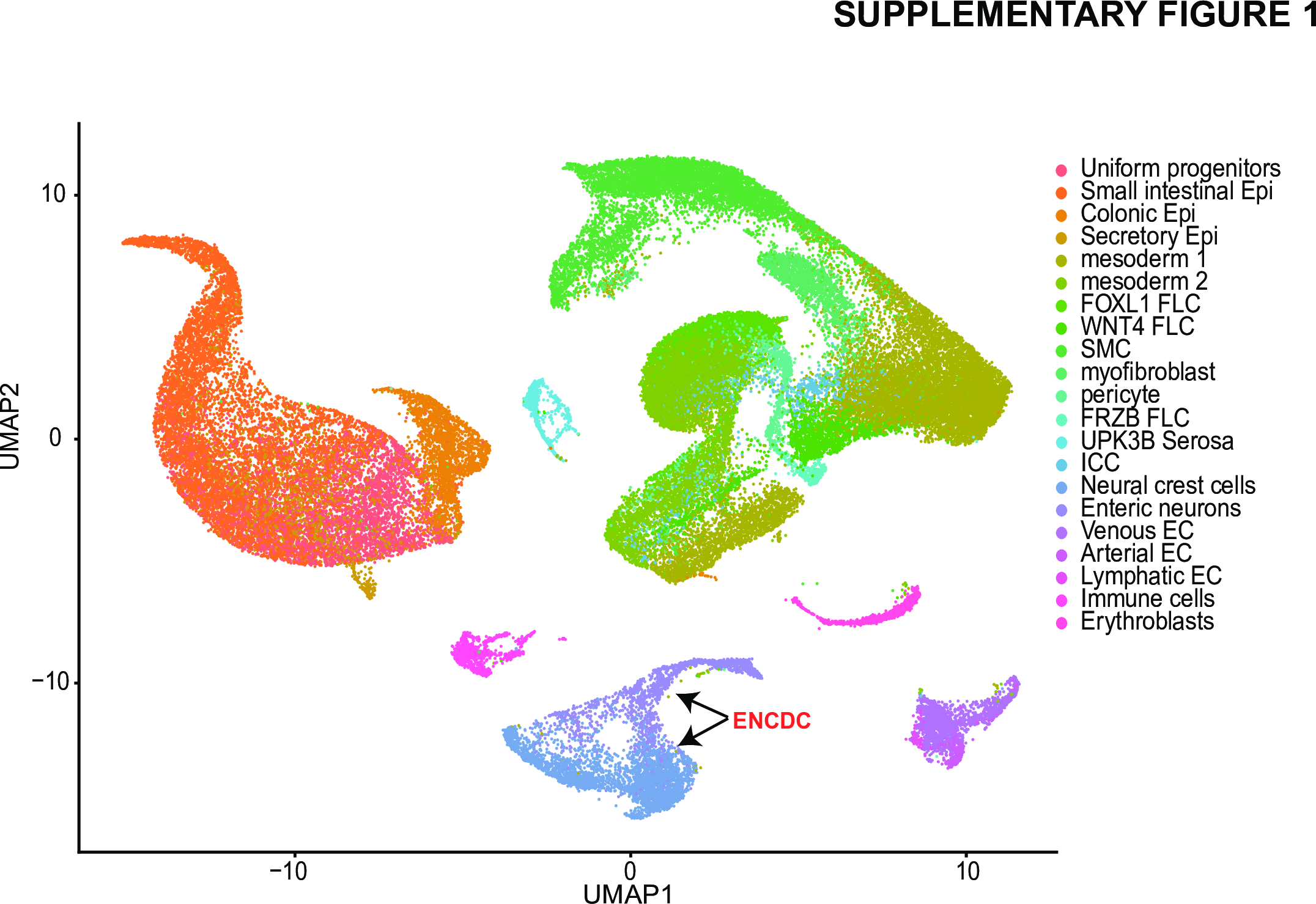

Supplement: S3 Fig — The diversity and distribution of 62,849 cells (UMAP, Uniform Manifold Approximation and Projection) in the developing human fetal gut at 6–11 weeks post-conception identifies 21 major cell types (Elmentaite, R et.al. Nature 2021). The enteric neural crest-derived cells (ENCDC) comprise 8% of the total and form 2 closely associated cell clusters. (TIF) [file pgen.1011030.s006.tif]

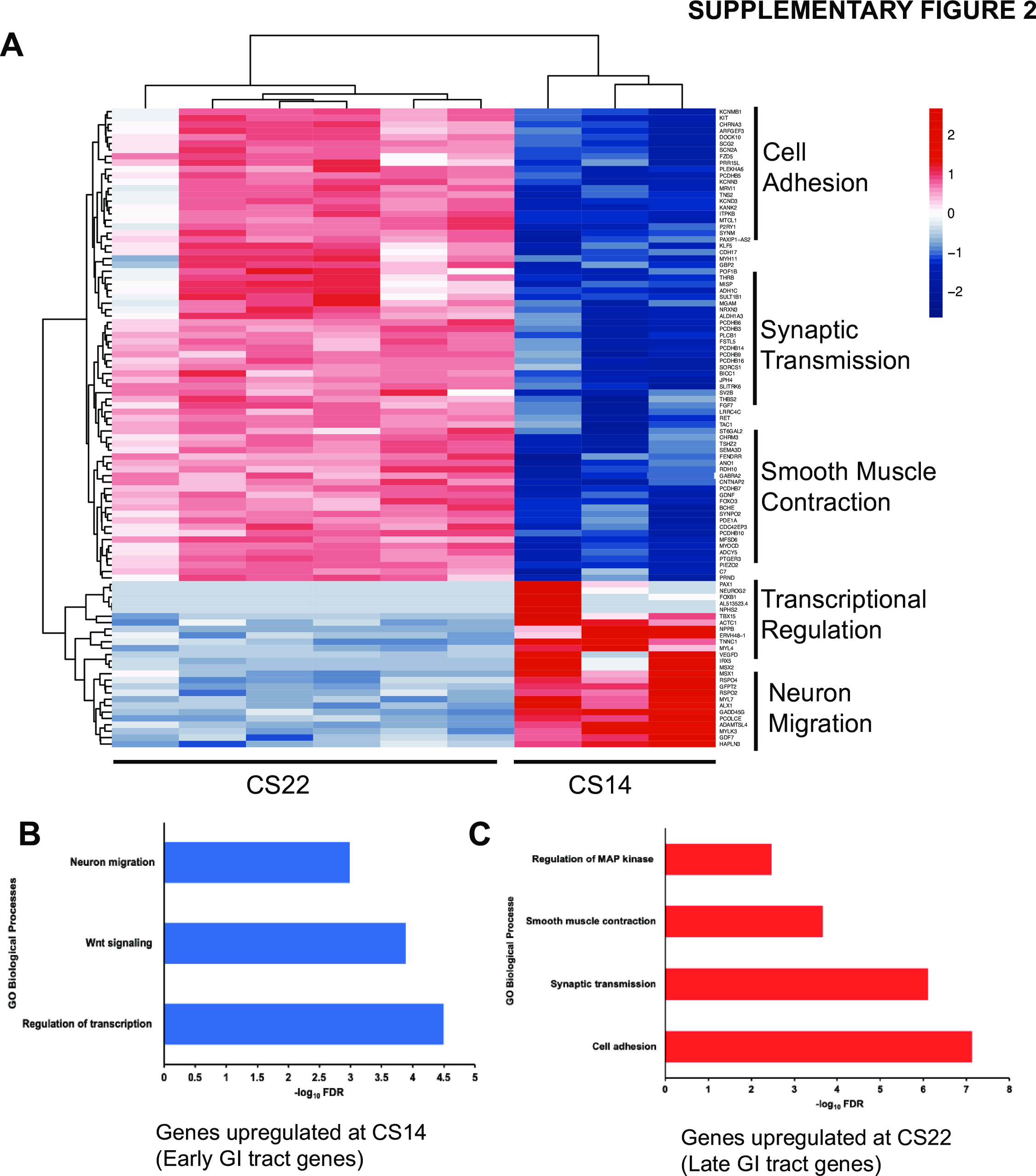

Supplement: S4 Fig — (A) Heatmap showing the top 100 differentially expressed genes at CS14 and CS22 stages of gut development. (B) At CS14, 657 genes show greater expression and are enriched for transcriptional regulation, neuronal migration and Wnt signaling functions, whereas (C) at CS22, 991 genes show greater expression and are enriched for organogenesis processes, such as, smooth muscle contraction, synaptic transmission along with cell adhesion and regulation of MAP kinase pathways. (TIF) [file pgen.1011030.s007.tif]
